# Antimicrobial Resistance Is Prevalent in *E. coli* and Other Enterobacterales Isolated from Public and Private Drinking Water Supplies in the Republic of Ireland

**DOI:** 10.3390/microorganisms11051224

**Published:** 2023-05-06

**Authors:** Maureen Daly, James Powell, Nuala H. O’Connell, Liz Murphy, Colum P. Dunne

**Affiliations:** 1 Department of Clinical Microbiology, University Hospital Limerick, V94 F858 Limerick, Ireland; 2 Biomedical Sciences Research Institute, University of Ulster, Coleraine BT52 1SA, UK; 3 Centre for Interventions in Infection, Inflammation & Immunity (4i), School of Medicine, University of Limerick, V94 T9PX Limerick, Ireland; 4 Public Health Laboratory, Raheen Business Park, V94 H9YE Limerick, Ireland

**Keywords:** antimicrobial resistance, AMR, environment, water, *Escherichia coli*

## Abstract

High levels of bacterial antimicrobial resistance (AMR) have been reported in many environmental studies conducted in Ireland and elsewhere. The inappropriate use of antibiotics in both human and animal healthcare as well as concentrations of residual antibiotics being released into the environment from wastewaters are thought to be contributing factors. Few reports of AMR in drinking water-associated microbes are available for Ireland or internationally. We analysed 201 enterobacterales from group water schemes and public and private water supplies, only the latter having been surveyed in Ireland previously. The organisms were identified using conventional or molecular techniques. Antimicrobial susceptibility testing for a range of antibiotics was performed using the ARIS 2X interpreted in accordance with EUCAST guidelines. A total of 53 *Escherichia coli* isolates, 37 *Serratia* species, 32 *Enterobacter* species and enterobacterales from seven other genera were identified. A total of 55% of isolates were amoxicillin resistant, and 22% were amoxicillin-clavulanic acid resistant. A lower level of resistance (<10%) was observed to aztreonam, chloramphenicol, ciprofloxacin, gentamicin, ceftriaxone and trimethoprim-sulfamethoxazole. No resistance to amikacin, piperacillin/tazobactam, ertapenem or meropenem was detected. The level of AMR detected in this study was low but not insignificant and justifies ongoing surveillance of drinking water as a potential source of antimicrobial resistance.

## 1. Introduction

The World Health Organisation (WHO, Geneva, Switzerland) emphasises antimicrobial resistance (AMR) as one of the top ten threats to global health, encouraging the urgent combined action across human health, animal health and the environment reflected in the “One Health” approach. Specifically, the WHO warn that we may be facing a post-antibiotic era whereby simple human infection may once again become life threatening, and routine medical procedures that rely on prophylactic antibiotic treatment may become impossible. Currently, it is estimated that 1.27 million global deaths annually are attributable to AMR [1], and it is projected that, by 2050, global annual deaths attributable to AMR will reach 10 million people [2]. This burden is carried most heavily in Sub-Saharan Africa (23.7 deaths per 100,000 population), while high-income countries are the least affected (13.0 deaths per 100,000 population) [1]. The Global Antimicrobial Resistance and Use Surveillance System (GLASS) was established by the WHO in 2015 to collate standardized data globally with respect to AMR in humans, the food chain and the environment, and surveillance of antimicrobial use. The GLASS data for antimicrobial resistance in bloodstream isolates in 2020 reflected Ireland’s higher resistance levels to ampicillin and ceftriaxone for *Escherichia coli* isolates when compared with the European median, yet resistance to ceftriaxone for *Klebsiella pneumoniae* was lower; ciprofloxacin resistance was lower in Ireland than the European median for both species [3]. Of the five global regions described by the GLASS (Africa, Europe, Eastern Mediterranean, South-East Asia and Western Pacific; insufficient data from the Americas), Europe had the lowest resistance to ampicillin, ceftriaxone and ciprofloxacin. Ireland is not currently enrolled in the GLASS antimicrobial consumption surveillance system, but overall, the global consumption of antibiotics has remained relatively stable in recent years, albeit there are some variations at regional and national levels. According to the GLASS, the highest antimicrobial consumption rates in 2020 were in the WHO Eastern Mediterranean Region (median Defined Daily Doses (DDD) per 1000 inhabitants: 31.8), double the rate observed in Europe, Africa and South-East Asia (all with median DDDs per 1000 inhabitants of 15.3 each) [3]. In Europe, the surveillance of sales volumes of veterinary antimicrobial agents is performed in the European Surveillance of Veterinary Antimicrobial Consumption (ESVAC) project in the European Medicines Agency. In their latest report, they describe a 46.5% decline in the aggregate volume of veterinary antimicrobials sold over the period from 2011 to 2021. The greatest reduction was for polymixins (79.5%) followed by 3rd and 4th generation cephalosporins (37.8%) and quinolones (34.7%) [4]. Ireland was the country with the twelfth highest volume of veterinary antimicrobials sold (in tons) in 2021 [4] despite being the country with the 3rd highest number of bovine animals, 6th highest number of sheep and 13th highest number of pigs in the EU [5].

The inappropriate use of antibiotics in both human and animal healthcare as well as residual concentrations of antibiotic being released into the environment from wastewaters are significant contributing factors in the emergence of antimicrobial resistance (AMR) [6]. In recognition of this, there has been considerable research regarding transmission of antimicrobial resistant organisms (AROs) in the clinical setting and, to a lesser extent, how misuse of antibiotics in human and animal health increases the incidence of AMR [7]. The natural environment is now also being acknowledged as a potentially important source of AMR and of ARO infection for both humans and animals [8]. This may be especially relevant to environments such as wastewater treatment plants, effluent from pharmaceutical companies, aquaculture facilities and animal husbandry facilities that have high bacterial loads often in addition to subtherapeutic levels of antibiotics that encourage ARO proliferation [9]. The transmission of such AROs can occur through direct contact within the environment and following consumption of food and drinking water [7]. Acquired antimicrobial resistance occurs because of the mutation or horizontal gene transfer of antimicrobial resistance genes often via mobile genetic elements, such as plasmids [8]. Extended Spectrum Beta Lactamase (ESBL) encoding genes are often located on plasmids, and aquatic environments are highlighted as important reservoirs and transmission routes for ESBL-producing enterobacterales [10]. Exogenous plasmid isolation is recommended as a means of detecting and quantifying plasmid-mediated antimicrobial resistance [11], but that is beyond the scope of this study.

Recognising the growing threat of AMR in the Republic of Ireland, its government launched a national interdepartmental AMR consultative committee in November 2014, and in October 2017, Ireland’s first national roadmap for tackling AMR was published. Ireland’s National Action Plans on Antimicrobial Resistance 2017–2020 and 2021–2025 (iNAP and iNAP2) defined how AMR should be managed in accordance with the One Health approach [12,13]. Among the factors highlighted as potential contributors to AMR were antimicrobials, resistant bacteria, biocides and heavy metals released into the environment through various routes. The WHO published a technical brief on water, sanitation, hygiene and wastewater management in 2020 [14]. It states that approximately two billion people worldwide obtain their drinking water from a source contaminated with faeces. The report encourages the improved management of wastewater treatment and sanitation systems to improve water quality, but it also recognised the importance of research on the risk of AMR transmission from drinking water. In Ireland, Carbapenemase Producing Enterobacterales (CPE) have been detected in hospital and municipal wastewater, which will inevitably result in the direct transmission of these organisms into the environment [15]. A link between exposure to drinking water contaminated with resistant *E. coli* and carriage of resistant *E. coli* in the human gastrointestinal tract has been established [16]. Notably, a large Canadian study found that, of 15,806 *E. coli* isolates obtained from 353,388 private well water samples, 10.5% were found to be resistant to one or more antibiotics, while 3.5% were found to be resistant to three or more classes of antibiotic [17]. However, neither North American study screened coliforms other than *E. coli* for AMR, perhaps underestimating the true level of AMR present.

With evidence that the prevalence of community-based infections attributable to multi-drug resistant organisms (MDROs) is rising, there is a need to examine all potential transmission routes, including AMR of coliforms and other non-fermenting Gram-negative bacteria [18]. Numerous recent studies on AMR in Irish recreational waters and wastewaters have been published [19,20,21,22]. In 2019, a point prevalence survey of AMR in the Irish environment included antibiotic susceptibility testing of isolates from seawaters, rivers and related sewage points [21]. The findings indicated a high prevalence of AROs, even from waters classified as excellent quality bathing waters. Published in February 2022, a separate study reported the presence of critically important AROs in Irish farm effluent with 82 AROs isolated from 96 samples tested [20]. Notably, no carbapenemase-producing organisms were isolated, but a significant level of ESBL, AmpC β-lactamase and flouroquinolone resistance was observed. These observations complement a German study that called for the establishment of a water safety program to include the monitoring of AMR in drinking water supplies [23]. This recommendation was based on levels of antibiotic residues and AROs determined for a drinking water supply in Germany over an 11-month period; specifically, AROs were detected at every site sampled. In Ireland, the Environmental Protection Agency (EPA) has warned that the quality of drinking water from private water supplies is a health risk. Approximately 180,000 households in Ireland have private well water sources, each with varying treatment systems in place [24]. These are not regulated under the Irish Drinking Water Regulations and are, therefore, unmonitored water supplies. Other private water supplies include 400 private group water schemes serving approximately 200,000 consumers and 1750 small private supplies serving commercial and public facilities, which are registered with local authorities [24]. Drinking water from any of these private supply sources is a potential health risk, affecting up to 20% of the Irish population or approximately 1 million people. This study attempts to provide a preliminary analysis of AMR in *E. coli* and other enterobacterales (indicator organisms of faecal contamination of drinking water) in public and private water supplies in Ireland during the first half of 2022.

## 2. Materials and Methods

Ethics: We conducted this study in accordance with ethical principles, even though formal ethical approval was not required as it did not involve the use of human or animal subjects.

Setting: In Ireland, food and water samples are monitored primarily through a network of six National Public Health Laboratories (PHLs) in Limerick, Cork, Galway, Waterford, Sligo and Dublin. Surveillance water samples are submitted from private and public supplies as well as targeted sampling by Environmental Health Officers. The six Public Health Laboratories were contacted in December 2021 and were invited to forward prospective consecutive *Escherichia coli* and other coliform isolates detected from drinking water. The isolates were forwarded to our clinical microbiology laboratory in the University Hospital Limerick. Our laboratory has extensive experience in characterising and monitoring AMR and AROs, and we have described a variety of outbreaks [25,26,27,28]. We are also continuously investigating and reporting new approaches and innovations in laboratory diagnostics [29,30,31,32,33,34], infection prevention and control interventions [35] and improvements in the clinical management of patients [36]. All six national laboratories participated in this study, providing a convenience sample of coliform isolates detected during routine testing of water samples. The laboratories supplied anonymous details for each isolate, which included (i) source location of the water sample, including the county of origin; (ii) whether the sample was taken from a private, public or group water supply; and (iii) identity of the organism as *E. coli* or coliform other than *E. coli*. In all cases, the procedure for collecting the water samples was according to the International Organization for Standardization (ISO) procedure 19458:2006. Water samples with a volume that ranged from 200 to 500 mL were collected into sterile containers containing sodium thiosulphate following cleaning of the water outlet and 2–3 min of flushing. They were transported at 2–8 °C and tested within 24 h of collection. Many of the specimens were collected by trained Environmental Health Officers (EHOs); for the remainder of cases, the specimens were collected by untrained individuals, and this is addressed in our study limitations. In all cases of private testing, detailed instructions were provided with the accompanying specimen containers and request form, but no assessment of the compliance with that procedure was performed. The samples were tested according to the ISO procedure 9308-2:2012 using the Colilert® method from IDEXX (Westbrook, Maine, ME, USA) for the enumeration of *Escherichia coli* and other coliform bacteria.

Identification: Each isolate was cultured on chromID CPS Elite (CPSE) chromogenic agar, where burgundy colonies have a presumptive identification of *Escherichia coli*. All other enterobacterales were identified using Sensititre ARIS 2X (Automated Reading and Incubation System, Mark II, Trek Diagnostics, Oakwood Village, OH, USA) and Matrix-Assisted Laser Desorption/Ionization-Time-of-Flight (MALDI-TOF, Bruker Daltonik (Bremen, Germany)) mass spectrometry (MS).

Susceptibility testing: Antibiotic susceptibility testing was performed for all isolates using Sensititre ARIS 2X in accordance with the European Committee on Antimicrobial Susceptibility Testing (EUCAST) guidelines v12.0, 2022. Susceptibility testing encompassed amoxicillin, co-amoxiclav, amikacin, aztreonam, chloramphenicol, ciprofloxacin, ertapenem, gentamicin, meropenem, ceftriaxone, piperacillin/tazobactam and trimethoprim/sulfamethoxazole. Isolates with susceptibility profiles suggestive of possible Extended Spectrum β-Lactamase or carbapenemase production were investigated further according to EUCAST guidance.

## 3. Results

**Figure 1:**
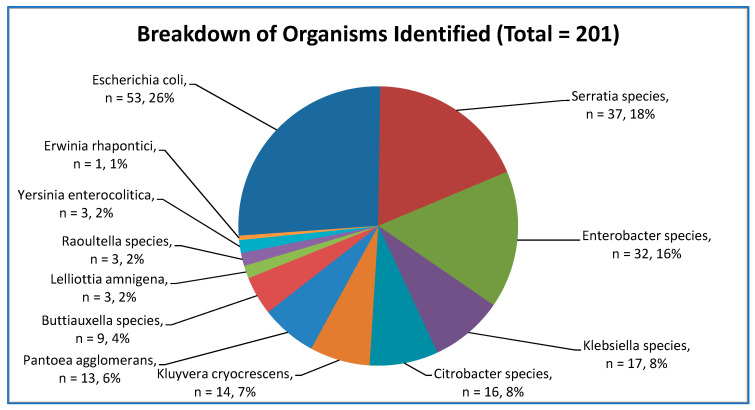
Organisms identified from water samples.

From January to May 2022, 201 eligible isolates were received, and four non-enterobacterales were excluded. A total of 186 (91%) were from private water supplies, 16 (8%) were from public water supplies, and 3 (1%) were from group water schemes. *E. coli* (n = 53, 26%) was the most commonly detected organism. The remaining isolates were *Serratia* sp. (n = 37), *Enterobacter* sp. (n = 32), *Klebsiella* sp. (n = 17), *Citrobacter* sp. (n = 16), *Kluyvera cryocrescens* (n = 14), *Pantoea agglomerans* (n = 13), *Buttiauxella* species (n = 9), *Lelliottia amnigena* (n = 3), *Erwinia rhaptonici* (n = 1), *Raoultella* sp. (n = 3) and *Yersinia enterolitica* (n = 3). Figure 1 details the organisms identified.

**Figure 2:**
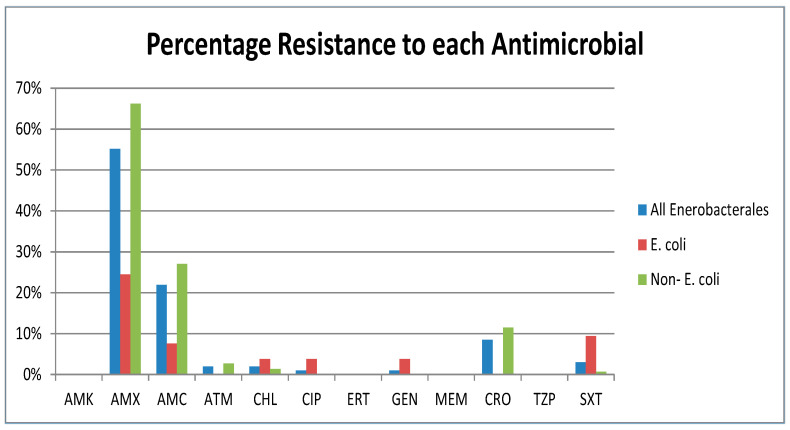
Percentage resistance of isolates (all, *E. coli* subset and remaining enterobacterales subset) to antimicrobials tested. AMK = Amikacin, AMX = Amoxicillin, AMC = Amoxicillin-clavulanic acid, ATM = Aztreonam, CHL = Chloramphenicol, CIP = Ciprofloxacin, ETP = Ertapenem, GEN = Gentamicin, MEM = Meropenem, CRO = Ceftriaxone, TZP = Piperacillin/Tazobactam, SXT = Trimethoprim-Sulfamethoxazole.

The overall percentage susceptibility of the isolates to each of the antibiotics tested is shown in Figure 2. A total of 55% of the isolates were amoxicillin resistant, and 22% were amoxicillin-clavulanic acid resistant. A lower level of resistance (<10%) was observed to aztreonam, chloramphenicol, ciprofloxacin, gentamicin, ceftriaxone and trimethoprim-sulfamethoxazole. No resistance to amikacin, piperacillin/tazobactam, ertapenem and meropenem was detected. Forty-five isolates showed reduced susceptibility to cefpodoxime and, therefore, were tested for ESBL production; however, all 45 were negative. Four of the 45 isolates displayed resistance to Cefoxitin on disk diffusion, indicating the possible presence of Amp C beta-lactamase resistance. The *E. coli* isolates (n = 53) showed greater resistance to chloramphenicol (4% vs. 1%), ciprofloxacin (4% vs. 0%) and gentamicin (4% vs. 0%) in comparison to the remainder of the enterobacterales in the study, and the inverse was found for amoxicillin (66% resistance for “other”, 25% for *E. coli*), amoxicillin-clavulanic acid (27% vs. 8%), aztreonam (3% vs. 0%) and ceftriaxone (11% vs. 0%).

In our study, a greater degree of resistance was noted in the enterobacterales other than *E. coli*. Of the 148 tested, just 49 (33%) showed no detectable resistance with 65% resistant to one antimicrobial and 2% resistant to two antimicrobials. The equivalent figures for the *E. coli* isolates in our study were 76% with no detectable resistance, 11% resistant to one antimicrobial and 13% resistant to two or more antimicrobials. Our data demonstrate that the isolates obtained from the public water supplies (n = 17) represented a higher level of resistance than those obtained from the private water supplies (n = 182). A total of 46% of the isolates from private sources had no detectable resistance compared with just 24% of the isolates from public water supplies. The small number of isolates from the public water supplies may have been due to better water treatment protocols and infrastructure, a better sampling technique by more experienced collectors or less water samples tested from these sources; the total number of water specimens tested in each laboratory is unknown.

**Figure 3:**
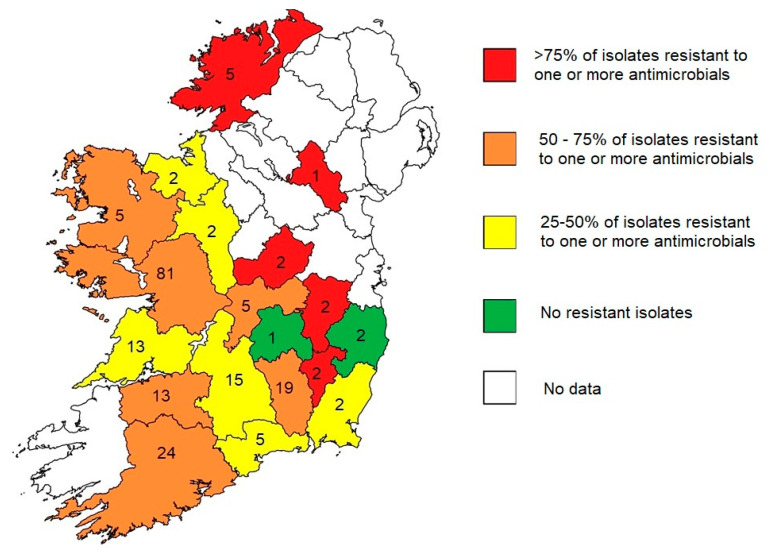
Geographical distribution of the isolates and resistance. The figures stated per county are the total number of isolates tested. The colour code for each county is the proportion of isolates showing any resistance. Note: The minimum denominator of 30 isolates was only achieved for one county, so comparison of resistance rates between counties is not possible. This chart is for illustrative purposes only.

The geographical distribution of the water samples is provided in Figure 3, detailing the number of samples per county and the percentage of isolates showing resistance to one or more antibiotics tested. Five counties showed resistance (to any antimicrobial) in >75% of the isolates tested, resistance was observed for 50–75% of the isolates from six counties, resistance was observed in 25–50% of the isolates from six counties, and the isolates from two counties were clear of detectable resistance. See Appendix A, Table A1 for a breakdown of the antimicrobial resistance detected per county. Note that the breakdown per county reduces the size of the denominator for these calculations with nine of the nineteen counties having less than five isolates tested. A minimum of 30 isolates is recommended for comparison of susceptibility percentages [37].

## 4. Discussion

This is the first study to determine the prevalence of AMR in public and private water supplies, including enterobacterales other than *E. coli*, across the Republic of Ireland. To achieve this, the isolates of *E. coli* and other enterobacterales detected during routine analysis of drinking water samples were collected from the HSE Public Health Laboratories across the country. Such studies have been performed internationally; however, comparison between studies is complex with many studies describing the prevalence of antimicrobial resistance genes rather than AROs. For example, a study from the USA found 6% of coliform-positive specimens contained either ESBL or carbapenemase genes [12], but many of the ESBL-producing organisms were not *E. coli*, and all of the carbapenemase-producing organisms were non-fermenting Gram-negative species. *E. coli* is the most commonly reported organism, and Table 1 shows a comparison of the resistance rates determined in our study and those available in the international literature, noting that the antimicrobial susceptibility testing techniques and interpretive guidelines varied across locations and over time, so caution should be exercised when comparing studies. Two previous studies of AMR in drinking water were performed in Ireland; both studies looked only at water from private groundwater wells, and both analysed *E. coli* [38,39]. One of these studies also tested *Pseudomonas aeruginosa* isolates, but no resistance was detected for that organism [38]. In our study, 25% of the *E. coli* isolates demonstrated some resistance (to any antimicrobial) compared with 67% of the other enterobacterales. This highlights the importance of including enterobacterales other than *E. coli* in AMR surveillance in this setting, a practice that has seldom been performed in previous similar studies. Similarly, we found a greater degree of resistant isolates from public water supplies in our study, a source of supply not included in the two previous AMR studies from this country. We recommend further AMR studies of this water source, which supplies the majority of households in this country.

**Figure A1:**
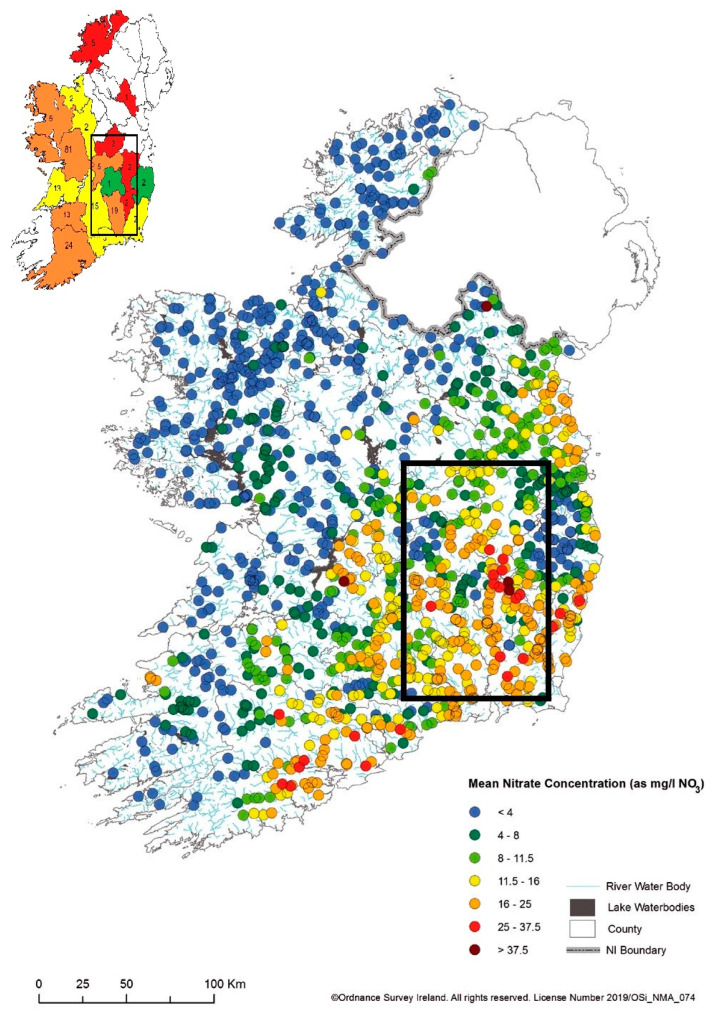
Mean nitrate concentrations in rivers during 2020 (modified). From the EPA report: Water quality monitoring report on nitrogen and phosphorus concentrations in Irish waters 2020 with the addition of a highlighted rectangle and inset Figure 3 with a similarly highlighted area.

It was noted that one of the regions where resistant bacteria were identified in our study was the South-East of Ireland, an area highlighted in Appendix A, Figure A1. In addition, in that image is an area identified by the Irish Environmental Protection Agency as having high nitrate concentrations in water sampled from Ireland’s rivers and lakes. It is well established that organic waste generated by farm animals and humans are a source of nitrate pollution [45], so while speculative, there may be a link between nitrate pollution and the spread of antimicrobial resistance in the environment. We suggest that further studies are warranted on this topic.

There are limited data available on the use of antimicrobials in agriculture in Ireland, but amoxicillin is commonly used in poultry and dairy farming [46], which could explain the very high levels of resistance observed in environmental water and sewage (both 97%) samples [21] and in our study of drinking water samples (55%). Amoxicillin is more commonly used in Irish hospitals in combination with clavulanate; this combination antibiotic accounts for 21% of all antibacterials used in a recent point prevalence study [47]. The European Centre for Disease Prevention and Control (ECDC) publishes a European Antimicrobial Resistance Surveillance Network (EARS-Net) report on clinical isolates annually. The data on clinical bacteraemia isolates of *E. coli* in Ireland in 2020 show 65%, 51%, 19% and 10% resistance to amoxicillin, co-amoxiclav, ciprofloxacin and gentamicin, respectively (n = 2795) [48]; the corresponding rates of resistance found among our *E. coli* isolates were 25%, 9%, 4% and 4% (n = 53).

The identification of three isolates of *Yersinia enterolitica* from three separate drinking water supplies was a significant finding of this study. This organism can be a significant human gastrointestinal pathogen with human infections associated commonly with the consumption of undercooked contaminated pork. Virulent strains are widespread in nature, but pathogenic strains have been identified from environmental water [49]. There are six recognised *Y. enterocolitica* biotypes (1A, 1B, 2, 3, 4 and 5) with biotype 1B considered highly virulent and biotype 1A classically considered non-pathogenic. More recently, it has been suggested that some *Y. enterocolitica* biotype 1A strains may cause human disease [50]. Therefore, our isolates will undergo whole-genome sequencing (WGS) to clarify their carriage of known virulence factors.

## 5. Conclusions

AMR is described by the United Nations as “one of the greatest threats we face as a global community” and “there is no time to wait” [51]. Structured and standardised international surveillance of AMR across all One Health pillars is critically important [6]. We focused on the environmental pillar of that strategy, and our study highlights the importance of drinking water as a potential source of AMR transmission. Penicillins are the second most commonly used antimicrobials in agriculture [4] and the most commonly used antimicrobials for humans in both community and hospital settings [52]. It is notable then that the agents with the most commonly detected antimicrobial resistance were amoxicillin (55% resistant) and amoxicillin-clavulanic acid (22%), both widely used penicillins across all sectors. Our results suggest that a significant proportion of Ireland’s human and animal populations may be ingesting enterobacterales that harbour resistance to these very important medicines, making them potentially less useful in the future. We recommend further AMR studies of bacteria identified from water sources, the inclusion of enterobacterales other than *E. coli* and also monitoring of all water sources including public water supplies in those surveillance programs. Failing this, some of the most potent vectors of AMR may go unrecognised.

## 6. Limitations

The isolates received for this study represented a convenience sample from laboratories around Ireland. As such, they are a subset of bacteria isolated by the referring laboratories. Similarly, not all positive Colilert wells from every sample were sub-cultured for the purposes of this study. One *E. coli* positive well and/or one coliform positive well only were cultured from each positive sample. Non-enterobacterales (e.g., non-fermenters, often with greater antimicrobial resistance levels than enterobacterales) were not included in this study. Some variation in sampling technique may have occurred; water samples from public water supplies were collected by professional Environmental Health Officers, while those from private supplies were performed by the individuals themselves. Comparison of the rates of resistance between regions was not possible due to study design and, specifically, the small sample size per county, which is evident in Figure 3. An accepted convention is for a minimum of 30 isolates per category in order to provide meaningful comparison [37].

## Figures and Tables

**Table 1:** Percentage resistance of *Escherichia coli* isolates. AMX = Amoxicillin, CIP = Ciprofloxacin, GEN = Gentamicin, SXT = Trimethoprim-Sulfamethoxazole, U/K = Unknown. ^¥^ Publication date. * “β-Lactam resistance”.

		Date ^¥^	Total Orgs	*E. coli*	AMX	CIP	GEN	SXT
**our results**	**Ireland**	**2023**	**201**	**53**	**25%**	**4%**	**4%**	**9%**
Andrade et al. [38]	Ireland	2023	66	54	13%	6%	0%	2%
O’Dwyer et al. [39]	Ireland	2017	42	42	14%	5%		
Coleman et al. [17]	Canada	2012	U/K	7063	4% *	0%	4%	
Larson et al. [40]	Peru	2023	U/K	117	28%			18%
Odonkor et al. [41]	Ghana	2022	83	23	22%	0%	0%	
Papandreou et al. [42]	Greece	2000	239	10	30%			0%
Rayasam et al. [43]	India	2019	U/K	104	100%	16%	2%	35%
Swedan et al. [44]	Jordan	2019	U/K	109	94%	16%		41%

## Data Availability

The data presented in this study are available on request from the corresponding author.

## Appendix A

**Table A1:** Type of water supply and antimicrobial resistance detected per county. GWS = Group Water Scheme. AMX = Amoxicillin, AMC = Amoxicillin-clavulanic acid, ATM = Aztreonam, CHL = Chloramphenicol, CIP = Ciprofloxacin, GEN = Gentamicin, CRO = Ceftriaxone, SXT = Trimethoprim-Sulfamethoxazole.

	GWS	Private	Public	Total	AMX	AMC	ATM	CHL	CIP	GEN	CRO	SXT	No Resistance	Resistance (Any)	Total	% Resistance (Any)
Carlow		2		2	2						2			2	2	100%
Kildare		2		2	2	1								2	2	100%
Monaghan		1		1	1									1	1	100%
Westmeath		2		2	2	1					1			2	2	100%
Donegal		5		5	4			1			1		1	4	5	80%
Kilkenny		4	15	19	12	4		1			2		7	12	19	63%
Cork		24		24	15	7		1	1	1	1	1	9	15	24	63%
Limerick		12	1	13	8	2					2	1	5	8	13	62%
Mayo		5		5	3								2	3	5	60%
Offaly		5		5	3		1				1		2	3	5	60%
Galway	1	80		81	44	21	2	1	1		3	3	37	44	81	54%
Roscommon		1	1	2	1	1							1	1	2	50%
Sligo		2		2	1					1		1	1	1	2	50%
Wexford		2		2	1						1		1	1	2	50%
Tipperary	1	14		15	7	4	1				2		8	7	15	47%
Waterford		5		5	1	1							3	2	5	40%
Clare		13		13	4	1					1		9	4	13	31%
Laois		1		1									1	0	1	0%
Wicklow		2		2									2	0	2	0%
Total	2	182	17	201	111	43	4	4	2	2	17	6	89	112	201	56%
